# Infiltration of peripheral immune cells into the olfactory bulb in a mouse model of acute nasal inflammation

**DOI:** 10.1016/j.jneuroim.2022.577897

**Published:** 2022-05-23

**Authors:** Hinami Asano, Sanae Hasegawa-Ishii, Ken Arae, Aki Obara, Geoffroy Laumet, Robert Dantzer, Atsuyoshi Shimada

**Affiliations:** a Pathology Research Team, Faculty of Health Sciences, Kyorin University, 5-4-1 Shimorenjaku, Mitaka-shi, Tokyo 181-8612, Japan; b Department of Immunology, Faculty of Health Sciences, Kyorin University, 5-4-1 Shimorenjaku, Mitaka-shi, Tokyo 181-8612, Japan; c Department of Analytical Chemistry, Faculty of Health Sciences, Kyorin University, 5-4-1 Shimorenjaku, Mitaka-shi, Tokyo 181-8612, Japan; d Department of Physiology, Michigan State University, 766 Service Rd, East Lansing, MI 48824, USA; e Department of Symptom Research, Division of Internal Medicine, The University of Texas MD Anderson Cancer Center, 1515 Holcombe, Blvd., Houston, TX 77030, USA

**Keywords:** Nasal inflammation, Olfactory nerve fibers, Peripheral immune cells, Meninges, Olfactory bulb, Resident microglia

## Abstract

Chronic nasal inflammation induces robust olfactory bulb (OB) atrophy in mice. Here we examined initial events that occur in the OB after bilateral intranasal administration of lipopolysaccharide, focusing on the olfactory nerve fibers and meninges. We analyzed the time course of OB and meninges inflammation using histological and biochemical approaches. Within 12 h, we observed increased chemokine expression and transient infiltration of peripheral immune cells into the OB, resulting in the development of pro-inflammatory status in the OB. Meningeal immunity was activated. Resident microglia produced anti-inflammatory cytokines within 24 h. These could be the initial events that lead to OB atrophy.

## 1. Introduction

A variety of potentially harmful agents in the environment, such as bacteria, viruses, and pollen, often enter the nasal cavity and cause inflammation in the olfactory epithelium (OE). According to epidemiological studies, patients with chronic rhinosinusitis (CRS) have a 1.4–2.6 times higher risk of psychiatric disorders, such as depression and anxiety (Erskine et al., 2017; Kim et al., 2016; Kim et al., 2019; Schlosser et al., 2016). In particular, the risk of depression is higher in CRS patients that do not have nasal polyps (Erskine et al., 2017; Kim et al., 2019; Schlosser et al., 2016). In addition, animal studies have revealed an association between permanent allergic rhinitis and anxiety-like behavior (Tonelli et al., 2009). Thus, nasal inflammation can impact brain function, however, the mechanisms connecting nasal inflammation to brain dysfunction remain unclear.

One route from the nose to the brain may be via olfactory nerve fibers wrapped with olfactory ensheathing cells. The cell bodies of the olfactory sensory neurons reside in the OE, and their axons extend through the cribriform plate to the olfactory bulb (OB), the most rostral part of the brain. The olfactory ensheathing cells envelop bundles of olfactory sensory neuron axons in the lamina propria of the olfactory mucosa and in the olfactory nerve layer (ONL) of the OB. In our previous study, intranasal administration of lipopolysaccharide (LPS) induced inflammation predominantly in the lateral side of the OE rather than the medial side, probably because the solution reached the turbinates on the lateral side and was drained by the airflow from the medial side (Hasegawa-Ishii et al., 2017). As the severity of OE inflammation increased, glial cells were activated, interneuron activity decreased around the glomeruli, and the number of dendro-dendritic synapses in the external plexiform layer (EPL) was reduced within 3 weeks, particularly on the lateral side of the OB (Hasegawa-Ishii et al., 2017). Given that olfactory sensory neurons located on the lateral side of the OE innervate the lateral part of the OB, inflammatory damage in the OE could affect the OB via the olfactory nerve fibers with ensheathing cells (neural route). Repeated administration of LPS to the nasal cavity for 10 weeks has been reported to induce atrophy of the OB (Hasegawa-Ishii et al., 2020; Hasegawa-Ishii et al., 2019). The outer three layers, namely the ONL, glomerular layer (GL), and superficial external plexiform layer (sEPL), were thinner on both the lateral and medial sides. In addition, the cessation of LPS administration led to the recovery of the OB (Hasegawa-Ishii et al., 2020, Hasegawa-Ishii et al., 2019).

Another potential route connecting the nose to the brain is the meninges. According to previous studies, when India ink is administered to the subarachnoid space of rats, it flows along the olfactory nerves to the nasal lymphatics, and then to the cervical lymph nodes (Yamazumi, 1989; Kida et al., 1993; Walter et al., 2006). This suggests that olfactory sensory neurons and the meninges communicate with each other (meningeal route). The meninges contain many immune cells, as well as lymphatic and blood vessels, that release a variety of factors into the cerebrospinal fluid (Rustenhoven et al., 2021; McMenamin et al., 2003; Louveau et al., 2015). Activation of meningeal cells mostly affects brain cell activities via various kinds of molecules (Derecki et al., 2010; Hasegawa-Ishii et al., 2016; Laumet et al., 2018). However, it is not known whether or how nasal inflammation affects meningeal immunity.

In the present study, we examined the initial events that would occur in the OB after intranasal LPS administration, focusing on the neural and meningeal routes. Our findings reveal the potential mechanisms underlying brain damage after nasal inflammation.

## 2. Methods

### 2.1. Animals

**Fig. 1.**
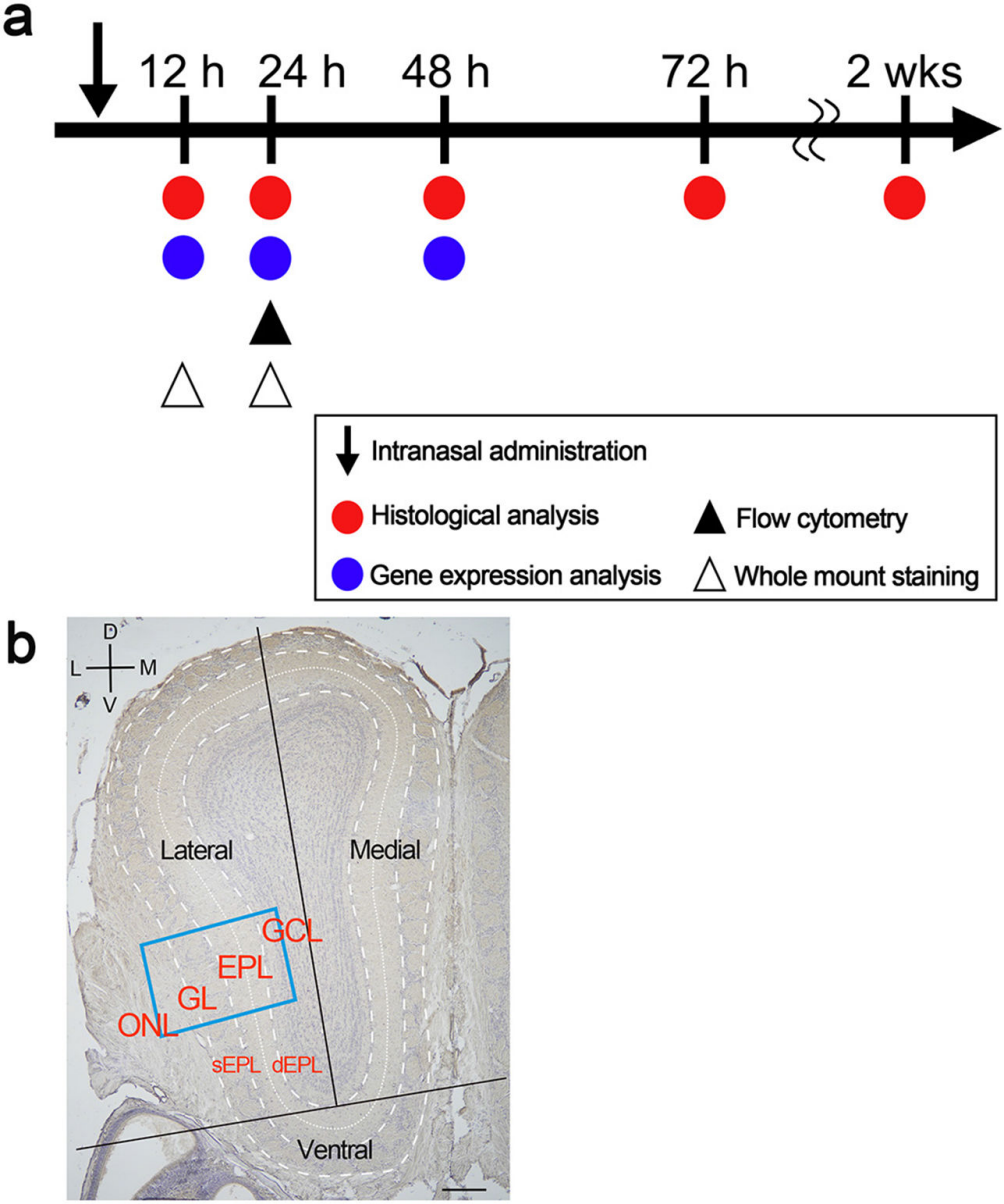
Experimental protocol. a Mice received a single bilateral intranasal administration of 10 μL of LPS or saline and were culled at 12, 24, 48, 72 h or 2 wks after intranasal administration. b The coronal section of the middle OB. For histological analysis, the middle OB was divided into lateral, medial, and ventral parts. The density of immune cells was determined in each layer, including the olfactory nerve layer (ONL), glomerular layer (GL), external plexiform layer (EPL), and granule cell layer (GCL). The EPL was further divided into the superficial EPL (sEPL), and deep EPL (dEPL). Photoimages in the Figs. 2-4 are magnified views of the rectangle in b. Scale bar: 200 μm.

Eight-week-old male C57BL/6JJmsSlc mice (Sankyo lab, Tokyo, Japan) were used in this study. To model acute nasal inflammation, mice were deeply anesthetized with isoflurane and received 10 μL physiological saline or LPS from *Escherichia coli* O55:B5 (Sigma, St. Louis, MO, USA) dissolved in physiological saline (1 mg/mL) into the bilateral nostrils at 20-min intervals (He et al., 2013; Mishima et al., 2021). Mice were perfused with 4% paraformaldehyde solution for histological preparation at 12, 24, 48, 72 h and 2 weeks (wks) after administration (saline48 h, LPS12 h, LPS24 h, LPS48 h, LPS72 h, and LPS2 wks, respectively). Given that immune cell infiltration, glial cell activation, and cytokine expression did not differ between saline-administered OB samples at 24 h, 48 h and 2 wks, the saline48 h samples were used as a control (Fig. 1a).

### 2.2. Histological preparation

Mice were anesthetized with ketamine (100 mg/kg body weight) and xylazine (10 mg/kg) and transcardially perfused with phosphate buffered saline (PBS), and then with 4% paraformaldehyde in PBS as the fixation solution. Their heads were decapitated and placed in the same fixation solution at 4 °C overnight. For each head, the lower jaw and the upper teeth were removed. The remaining skull and brain were placed en bloc in 2× K-CX (Falma, Tokyo, Japan) for 6 h at room temperature for decalcification, and then washed with floating water for more than 6 h. The skull and brain were cryoprotected with 20% sucrose (wt/vol) at room temperature overnight, embedded in OCT compound (Sakura Finetek, Torrance, CA, USA), and frozen in 100% ethanol cooled with dry ice. Frozen blocks were stored at −80 °C until use.

For the histological preparation of the whole mount meninges, mice were deeply anesthetized with ketamine and xylazine, and transcardially perfused with PBS. The heads were collected, the skin and flesh were detached from the skull, and the jaws and brains were removed. Then, the skullcaps were post fixed with the same fixation solution at 4 °C for 1 week. The meninges were carefully peeled off from the skullcap as one piece and placed into PBS, as previously described (Laumet et al., 2018).

### 2.3. Immunostaining

Olfactory tissues were coronally cut into 16 μm slices using a cryostat, mounted on slide glasses, dried, and stored at −30 °C until use. The sections were rehydrated with TBST [10 mmol/L Tris-HCl (pH 7.4) and 100 mmol/L NaCl with 0.1% Tween 20]. For antigen retrieval, sections were incubated in citrate buffer (pH 6.0) or in Tris-EDTA buffer (pH 9.0) at 80 °C for 30 min, and cooled to room temperature. Sections were blocked with blocking buffer [5% normal horse serum for immunohistochemistry or 5% normal donkey serum (*v*/v) for immunofluorescence in TBST] at room temperature for 1 h, and incubated with primary antibodies diluted in blocking buffer overnight. The primary antibodies used in the present study are listed in Table 1. Sections were then incubated with host-matched secondary antibodies (ImmPress anti-rabbit, anti-rat, anti-goat IgG, Vector Laboratories, Burlingame, CA, USA) at room temperature for 1 h and stained with ImmPACT DAB (Vector Laboratories). Nuclei were stained with Carrazzi’s hematoxylin for 1 min. Slices were cleared and coverslipped with HSR (Sysmex, Kobe, Japan). Sections were examined using an Eclipse Ci-L microscope equipped with a digital camera control unit (DS-Fi3/NIS-Elements; Nikon, Tokyo, Japan).

For double immunofluorescence staining, Alexa Fluor 568- conjugated or 488-conjugated donkey anti-species IgGs (Thermo Fisher Scientific, Waltham, MA, USA) were used as secondary antibodies (1:300). Nuclei were counterstained with 4′,6-diamidino-2-phenylindole (DAPI). The sections were coverslipped with fluorescence mounting medium (Dako, Glostrup, Denmark). Images were obtained by fluorescence microscopy with structured illumination using z-stack with a step size of 1 μm (BZ-X710; Keyence, Osaka, Japan).

For immunostaining of the whole mount meninges, the meninges were placed in TBST for 10 min, blocked with blocking buffer (5% normal donkey serum in TBST) at room temperature for 1 h, and incubated with primary antibodies diluted in blocking buffer at room temperature overnight. Then, the meninges were incubated with host-matched secondary antibodies for 1 h and coverslipped with fluorescence mounting medium (Dako). For antigen retrieval, meninges were placed in citrate buffer (pH 6.0) or Tris-EDTA buffer (pH 9.0) at 80 °C for 30 min.

### 2.4. Image analyses and morphometry

All samples were randomly numbered so that the experimenter who performed the analyses was a blind to sample identities.

#### 2.4.1. Cell density

Sections from the middle OB (defined in Hasegawa-Ishii et al., 2020), which is known to be atrohphied by chronic nasal inflammation (Hasegawa-Ishii et al., 2017; Hasegawa-Ishii et al., 2019; Hasegawa-Ishii et al., 2020), were stained with antibodies for CCR2, Ly6G, CD3e, and CD45R, and nuclei were stained with Carrazzi’s hematoxylin. Since infiltrating cells were not evenly distributed in the OB, the cell density was analyzed separately in three OB regions, namely the lateral, medial and ventral. The OB was divided into the lateral and medial OB by the center line drawn along the core of the OB. To divide the ventral OB, the line was drawn vertically to the center line at the most ventral part of the GCL. The OB was further divided into four layers–the ONL, GL, EPL, and GCL–in the lateral and medial OB, or into three layers–the ONL, GL, and EPL–in the ventral OB (Fig. 1b). In each region, the immunopositive cells with nuclei were counted. The area of each region was measured by counting the number of pixels enclosed by the boundaries and converting this to an area (mm^2^) using Photoshop software (Adobe Systems Inc. San Jose, CA, USA). Cell density was calculated by dividing the number of cells by the layer area (cells/mm^2^).

#### 2.4.2. sEPL area

In our previous study, the sEPL comprised around 50% of the total EPL when the EPL was divided into the sEPL and deep EPL (dEPL) using calretinin immunofluorescence (Hasegawa-Ishii et al., 2020). Thus, in the present study, the superficial half of the EPL was referred to as the sEPL, while the other half was defined as the dEPL.

### 2.5. Flow cytometry

Fresh OBs were collected at 24 h post saline or 24 h post LPS, minced in enzyme mix (Multi Tissue Dissociation kit 1, Miltenyi Biotec, Bergisch Gladbach, Germany) using scissors, and further minced and digested by running the gentle MACS Program 37C_Multi_F for 45 min (Posel et al., 2016). OB cells were collected after centrifugation, resuspended in buffer, and filtered using 70 μm nylon mesh. Red blood cells and debris were removed using Debris Removal Solution (Miltenyi).

For the flow cytometry of myeloid cells, the dissociated OB cells were incubated on ice for 20 min with anti-mouse CD16/CD32 monoclonal antibody (mAb) in fluorescence-activated cell sorting (FACS) buffer for FcR blocking. The cells were then incubated on ice for 30 min with a mixture of Ab cocktails containing Allophycocyanin (APC)-conjugated anti-mouse/human CD11b mAb (M1/70; BioLegend), fluorescein isothiocyanate (FITC)-conjugated anti-mouse CD45 mAb (30-F11; Bio-Legend), phycoerythin (PE)-conjugated anti-mouse CCR2 mAb (#475301; R&D Systems, Minneapolis, MN, USA), PE/Cyanin (Cy)7-conjugated anti-mouse TMEM119 mAb (V3RT1GOsz; Invitrogen-Thermo Fisher Scientific), pridinin chlophyll protein (PerCp)-conjugated anti-mouse Ly6G/Ly6C (Gr-1) mAb (RB6-8C5; BioLegend) and eBioscience^™^ Fixable Viability Dye eFluor^™^ 780. For the flow cytometry of lymphocytes, the dissociated OB cells were incubated (20 min on ice) with anti-mouse CD16/CD32 mAb (93; BioLegend, SanDiego, CA, USA) in FACS buffer (PBS(−) containing 2% fetal calf serum) for FcR blocking. The cells were then incubated on ice for 30 min with a mixture of antibody cocktails containing APC-conjugated anti-mouse/human CD45R/B220 mAb (RA3-6B2; BioLegend), FITC-conjugated anti-mouse CD45 mAb (30-F11; BioLegend), PE/Cy7-conjugated anti-mouse CD3e mAb (145-2C11; BioLegend) and eBioscience^™^ Fixable Viability Dye eFluor^™^ 780 (eBioscience-Thermo Fisher Scientific). After washing, the cell suspension was analyzed on a FACS Aria II Cell Sorter (BD Bio-sciences, Franclin Lakes, NJ, USA) using BD FACS Diva software (v6.1.3, BD Biosciences) and FlowJo software (v10.5.3, BD Biosciences). In each experiment, four OBs from two saline24 h and two LPS24 h mice were analyzed individually, three or four independent experiments were performed.

### 2.6. Relative quantitative RT-PCR assay

Brains from saline12 h, LPS12 h, saline24 h, LPS24 h, saline48 h, and LPS48 h groups of mice were collected, and the OBs were dissected on ice under a stereomicroscope, snap frozen in liquid nitrogen, and stored at −80 °C until use.

Total RNA was isolated from the frozen OBs using a NucleoSpin^®^ RNA kit (MACHEREY-NAGEL GmbH & Co. KG, Valencienner, Duren, Germany) according to the manufacturer’s instructions. RNA quantity was checked by examining absorbance values. First-strand cDNA was synthesized from 200 ng of total RNA in 5 × First-strand buffer containing 0.1 M DDT, RNase OUT, Super-Script III and RNase H (Thermo Fisher Scientific). Reverse transcription was performed at 65 °C for 5 min, 25 °C for 5 min, 50 °C for 60 min, 70 °C for 15 min, and 37 °C for 20 min (Thermo Fisher Scientific). Real-time PCR was performed using TaqMan Gene Expression Assays (Applied Biosystems-Thermo Fisher Scientific, Table 2), according to the manufacturer’s instructions, in a total volume of 20 μL with 40 cycles (95 °C for 3 s and 60 °C for 30 s) using the 7500 Fast Real-Time PCR System (Applied Biosystems-Thermo Fisher Scientific). Transcript levels were normalized to those of *Gapdh* or *Hprt* genes. Data are expressed as the relative fold change using the 2^-(ΔΔCT) calculation method. Error bars are based on the SD of the ΔΔCT values [2^-(ΔΔCT +/− SD of ΔΔCT)].

### 2.7. Statistical analysis

Statistical analyses were performed using Statistica software (Dell Software, Round Rock, TX, USA).

#### 2.7.1. Histological analysis

Three sections from the middle OB were selected per mouse for statistical analysis. The mean cell density was compared between saline48 h, LPS12 h, LPS24 h, LPS48 h, LPS72 h and LPS2 wks groups in the lateral, medial and ventral side of the OB, using a two-way analysis of variance (ANOVA, two main effects of treatment: saline, LPS12 h, LPS24 h, LPS48 h, LPS72 h, and LPS2 wks, and layers: ONL, GL, EPL, and GCL), followed by Tukey’s HSD post hoc tests for multiple comparisons. A p-value <0.05 was considered to indicate a significant difference. Values are reported as the means ± sem.

#### 2.7.2. Flow cytometry analysis

Within the myeloid cell subsets, the numbers of TMEM119-CD11b + CCR2+ and TMEM119-CD11b + Gr-1+ cells were compared between LPS24 h and saline24 h groups. Within the lymphocyte subsets, CD3e + or B220+ cells were compared between LPS24 h and saline24 h groups. Results were statistically analyzed using student *t*-test. A p-value <0.05 indicated a significant difference.

#### 2.7.3. Gene expression analysis

Gene expression data are reported as relative fold change using the 2^-(ΔΔCT) calculation method. Values of ΔCT were statistically analyzed using a two-way ANOVA (two main effects of treatment: saline and LPS; three time points: 12 h, 24 h, and 48 h), followed by Tukey’s HSD post hoc tests for multiple comparisons. A p-value <0.05 indicates a significant difference.

## 3. Results

### 3.1. Infiltration of CCR2-immunopositive cells into the olfactory bulb

**Fig. 2.**
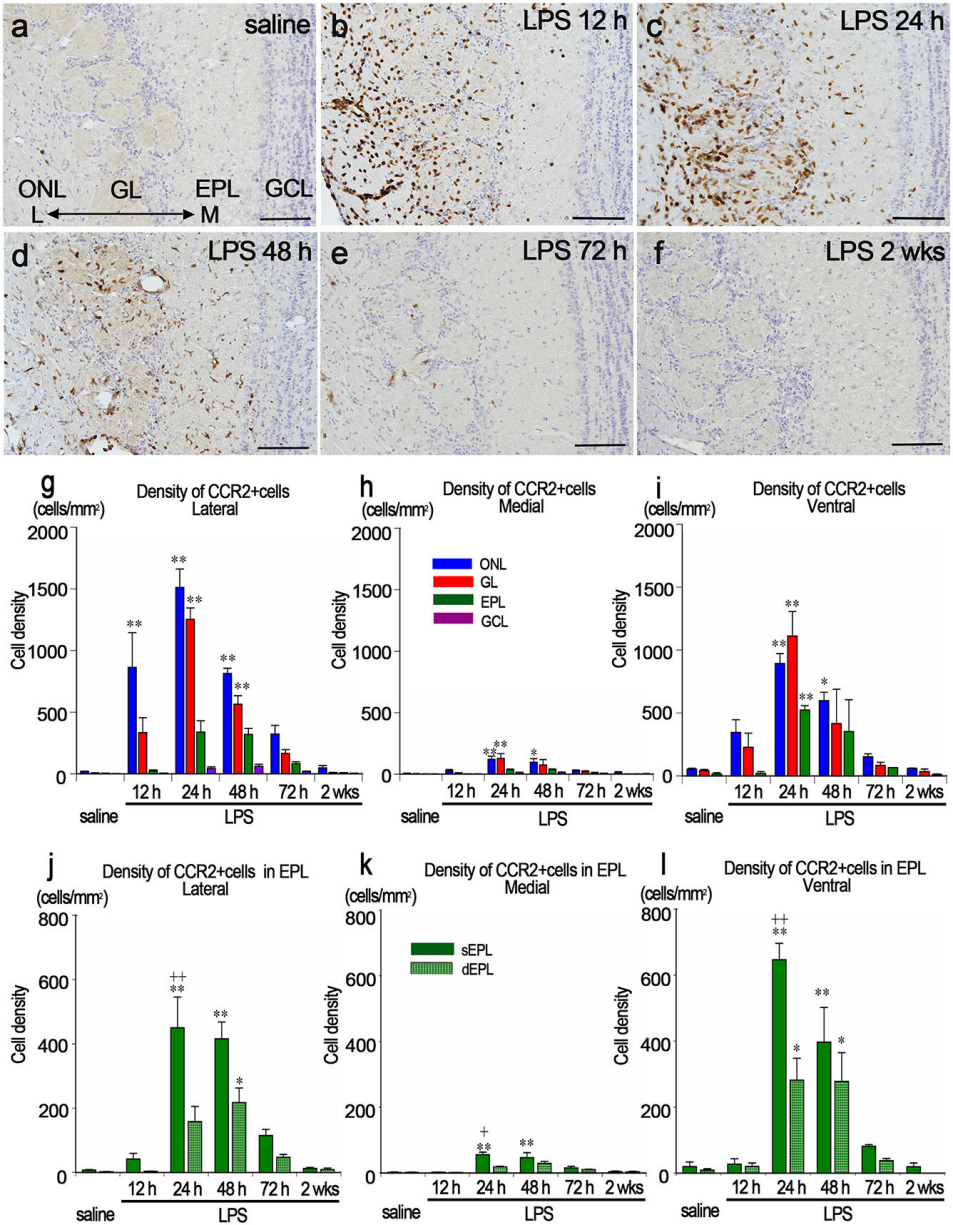
Infiltration of CCR2-immunopositive cells into the OB. a-f Immunohistochemistry for CCR2 in the lateral side of the OB in saline (a), LPS12 h (b), LPS24 h (c), LPS48 h (d), LPS72 h (e), and LPS2 wks (f) groups. Scale bars: 100 μm. g-i The density of CCR2-immunopositive cells in the lateral (g), medial (h), and ventral (i) sides of the OB (cells/mm^2^). *p < 0.05, **p < 0.01, compared with the saline control. CCR2-immunopositive cells particularly infiltrated the lateral side of the OB. n = 3 for each group, j-l The density of CCR2-immunopositive cells that infiltrated the EPL of the OB in the lateral (j), medial (k) and ventral (l) side of the OB (cells/mm^2^). *p < 0.05, **p < 0.01, compared to the saline controls. ^++^ p < 0.01, compared with dEPL. CCR2-immunopositive cells infiltrated the sEPL more than they did the dEPL (j-l). n = 3 for each group.

CCR2-immunopositive cells were not present in the OB in the saline controls, but infiltrated the OB after intranasal LPS administration (Fig. 2a-i). At 12 h after LPS administration, 864 ± 282 CCR2-immunopositive cells/mm^2^ infiltrated the lateral side of the ONL, many of which were located along the olfactory nerve fibers or around blood vessels (Fig. 2b, g). At 24 h post LPS, 1512 ± 150 and 1253 ± 93 CCR2-immunopositive cells/mm^2^ infiltrated the lateral side of the ONL and GL. In addition, CCR2-immunopositive cells infiltrated the lateral side of the sEPL (Fig. 2c, g, j). At 48 h post LPS, 814 ± 42 and 565 ± 69 CCR2-immunopositive cells/mm^2^ infiltrated the lateral side of the ONL and GL. CCR2-immunopositive cells infiltrated not only the sEPL, but also the dEPL (Fig. 2d, g, j). At 72 h post LPS, CCR2-immunopositive cells were still visible in the lateral sides of the ONL, GL, and sEPL, but their number did not differ significantly from that of the saline control (Fig. 2e, g, j). At 2 wks after LPS administration, almost no CCR2-immunopositive cells were found in any layers of the OB (Fig. 2f, g, j).

Much fewer CCR2-immunopositive cells infiltrated the medial side of the OB than the lateral side, but significantly more CCR2-immunopositive cells infiltrated the ONL at 24 h and 48 h, the GL at 24 h, and sEPL at 24 h and 48 h post LPS than in the saline controls (Fig. 2h, k).

In the ventral side of the OB, significantly more CCR2-immunoreopositive cells infiltrated the ONL at 24 h and 48 h, and the GL and EPL at 24 h post LPS than in the saline controls. CCR2-immunopositive cells particularly appeared in the sEPL rather than the dEPL, similarly to the lateral side (Fig. 2i, l).

### 3.2. Infiltration of Ly6G-immunopositive cells into the olfactory bulb

**Fig. 3.**
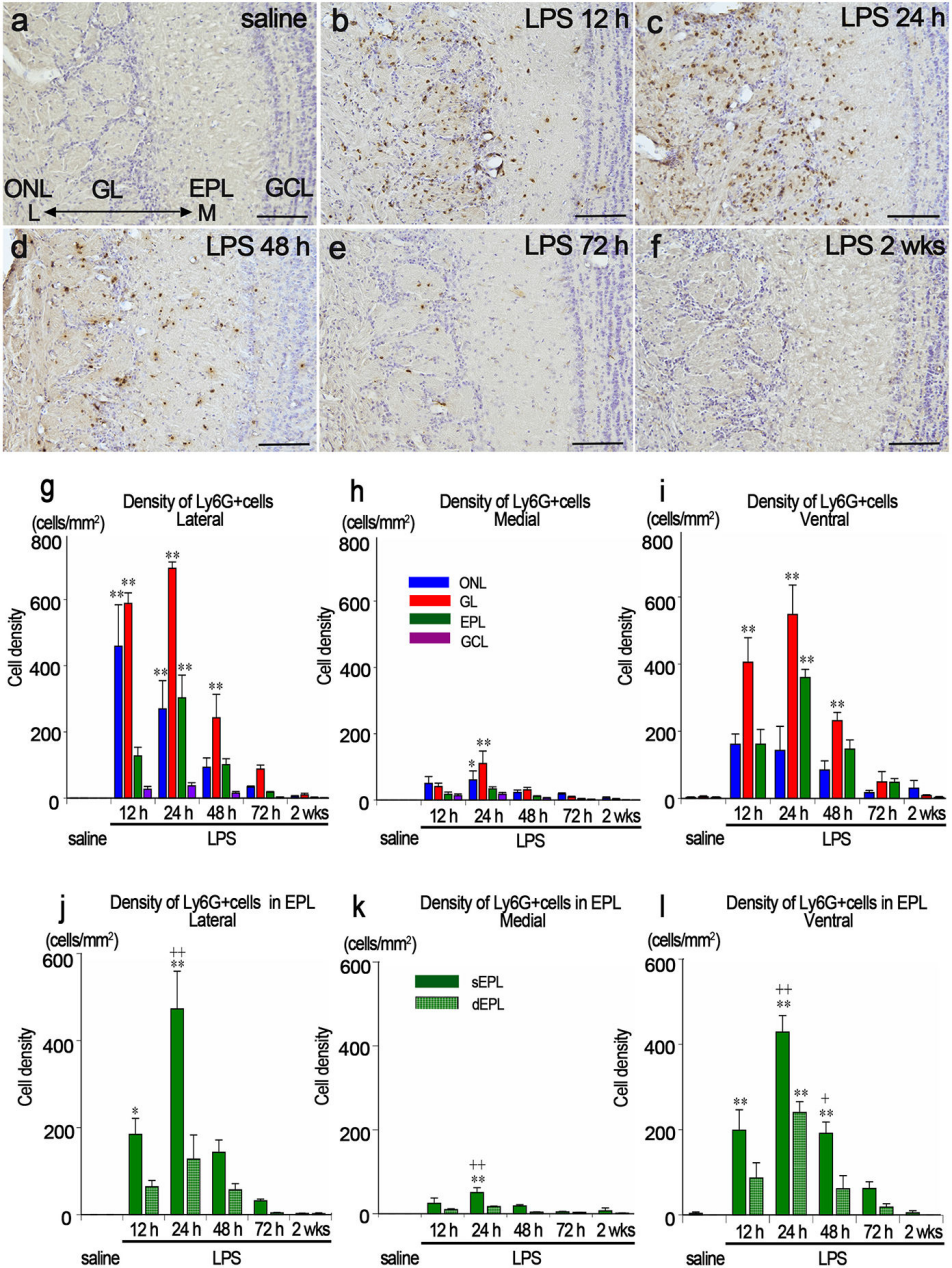
Infiltration of Ly6G-immunopositive cells into the OB. a-f Immunohistochemistry for Ly6G in the lateral side of the OB in saline (a), LPS12 h (b), LPS24 h (c), LPS48 h (d), LPS72 h (e) and LPS2 wks (f) groups. Scale bars: 100 μm. g-i The density of Ly6G-immunopositive cells in the lateral (g), medial (h), and ventral (i) sides of the OB. *p < 0.05, **p < 0.01, compared with the saline controls. Ly6G-immunopositive cells particularly infiltrated the lateral side of the OB. n = 3 for each group with the exception of n = 4 for the LPS72 h group. j-l The density of Ly6G-immunopositive cells that infiltrated the EPL of the OB in the lateral (j), medial (k), and ventral (l) sides of the OB. *p < 0.05, **p < 0.01, compared with the saline controls. ^++^ p < 0.01, compared with the dEPL. Ly6G-Immunopositive cells infiltrated the sEPL more than they did the dEPL (j-l). n = 3 for each group with the exception of n = 4 for the LPS72 h group.

Ly6G-immunopositive cells were not distributed in the OB in the saline control, but infiltrated the OB after LPS administration (Fig. 3a-i). At 12 h post LPS, 459 ± 125 and 588 ± 32 Ly6G-immunopositive cells/mm^2^ infiltrated the lateral side of the ONL and GL, respectively, many of which were located around blood vessels, but not along olfactory nerve fibers (Fig. 3b, g). At 24 h post LPS, 270 ± 85, 694 ± 19, and 303 ± 69 Ly6G-immunopositive cells/mm^2^ infiltrated the lateral sides of the ONL, GL, and EPL, respectively (Fig. 3c, g). In the EPL, Ly6G-immunopositive cells were particularly distributed in the sEPL (Fig. 3j). At 48 h post LPS, there were many small Ly6G-immunopositive debris that had no nuclei in the lateral sides of the GL and EPL (Fig. 3d, g). These debris were engulfed by microglia (Supplementary Fig. 1). At 72 h post LPS, a few Ly6G-immunopositive cells remained in the GL, but their number did not differ significantly from that of the saline control (Fig. 3e, g). At 2 wks post LPS, almost no Ly6G-immunopositive cells were found in any layers of the OB (Fig. 3f, g).

Much fewer Ly6G-immunopositive cells infiltrated the medial side of the OB than the lateral side, but significantly more Ly6G-immunopositive cells infiltrated the ONL and GL at 24 h post LPS than in the saline controls (Fig. 3h).

In the ventral side of the OB, there were significant differences in the GL at 12 h, 24 h and 48 h and in the EPL at 24 h post LPS (Fig. 3i). The densities of Ly6G-immunopositive cells were much higher in the sEPL than in the dEPL (Fig. 3l).

### 3.3. Infiltration of lymphocytes into the olfactory bulb

**Fig. 4.**
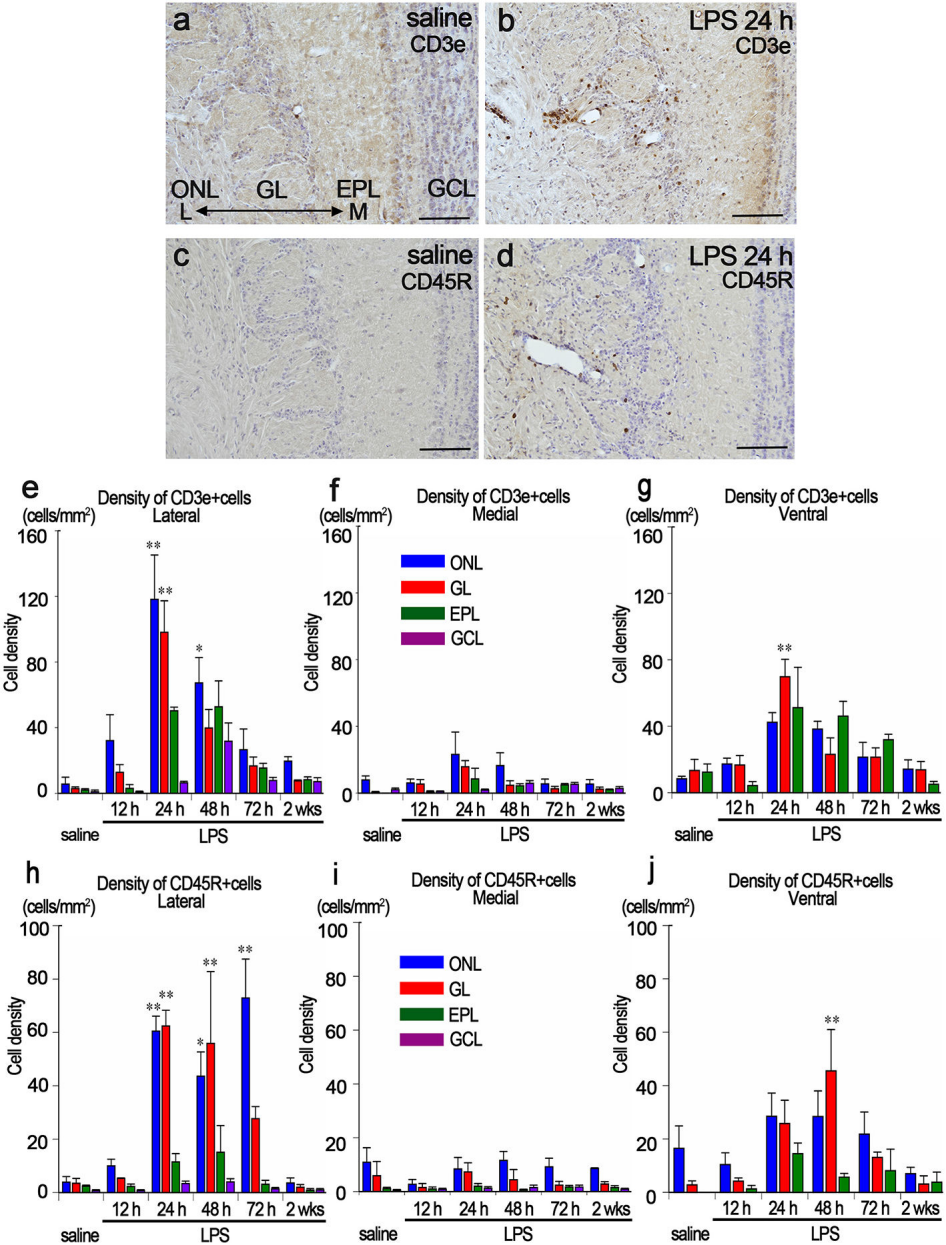
Infiltration of lymphocytes into the OB. a, b Immunohistochemistry for CD3e in the lateral side of the OB in saline (a) and LPS24 h groups (b). c, d Immunohistochemistry for CD45R in the lateral side of the OB in saline (c) and LPS24 h (d) groups. Scale bars: 100 μm. e-g The density of CD3e-immunopositive cells in the lateral (e), medial (f), and ventral (g) sides of the OB. *p < 0.05, **p < 0.01, compared with the saline controls. CD3e-immunopositive cells particularly infiltrated the lateral side of the OB. n = 3 for each group. h-j The density of CD45R-immunopositive cells in the lateral (h), medial (i), and ventral (j) sides of the OB. *p < 0.05, **p < 0.01, compared with the saline controls. CD45R-immunopositive cells particularly infiltrated the lateral side of the OB. n = 3 for each group.

CD3e-immunopositive cells were not distributed in the OB in the saline controls or at 12 h post LPS, but particularly infiltrated the lateral side of the OB at 24 h and 48 h post LPS (Fig. 4a, b, e). At 24 h post LPS, 118 ± 27 and 98 ± 19 CD3e-immunopositive cells/mm^2^ infiltrated the lateral sides of the ONL and GL, respectively, many of which were located around blood vessels (Fig. 4b). The number of CD3e-immunopositive cells was still higher in the ONL at 48 h post LPS (Fig 4e). At 72 h and 2 wks post LPS, there were no significant differences in the density of CD3e-immunopositive cells between saline- and LPS-treated mice (Fig. 4e).

In the medial side of the OB, there were no significant differences in the density of CD3e-immunopositive cells in any layers between saline- and LPS-treated mice (Fig. 4f). In the ventral side of the OB, significantly more CD3e-immunopositive cells infiltrated the GL at 24 h post LPS than in the saline controls, but there were no other significant differences (Fig. 4g).

CD45R-immunopositive cells markedly infiltrated the lateral side of the OB at 24 h, 48 h and 72 h post LPS, many of which were located around blood vessels (Fig. 4c, d, h). In the medial side of the OB, there were no significant differences in the density of CD45R-immunopositive cells between saline- and LPS-treated mice (Fig. 4i). In the ventral side of the OB, significantly more CD45R-immunopositive cells infiltrated the GL at 48 h post LPS than in the saline controls, but there were no other significant differences (Fig. 4j).

### 3.4. Flow cytometry analysis

**Fig. 5.**
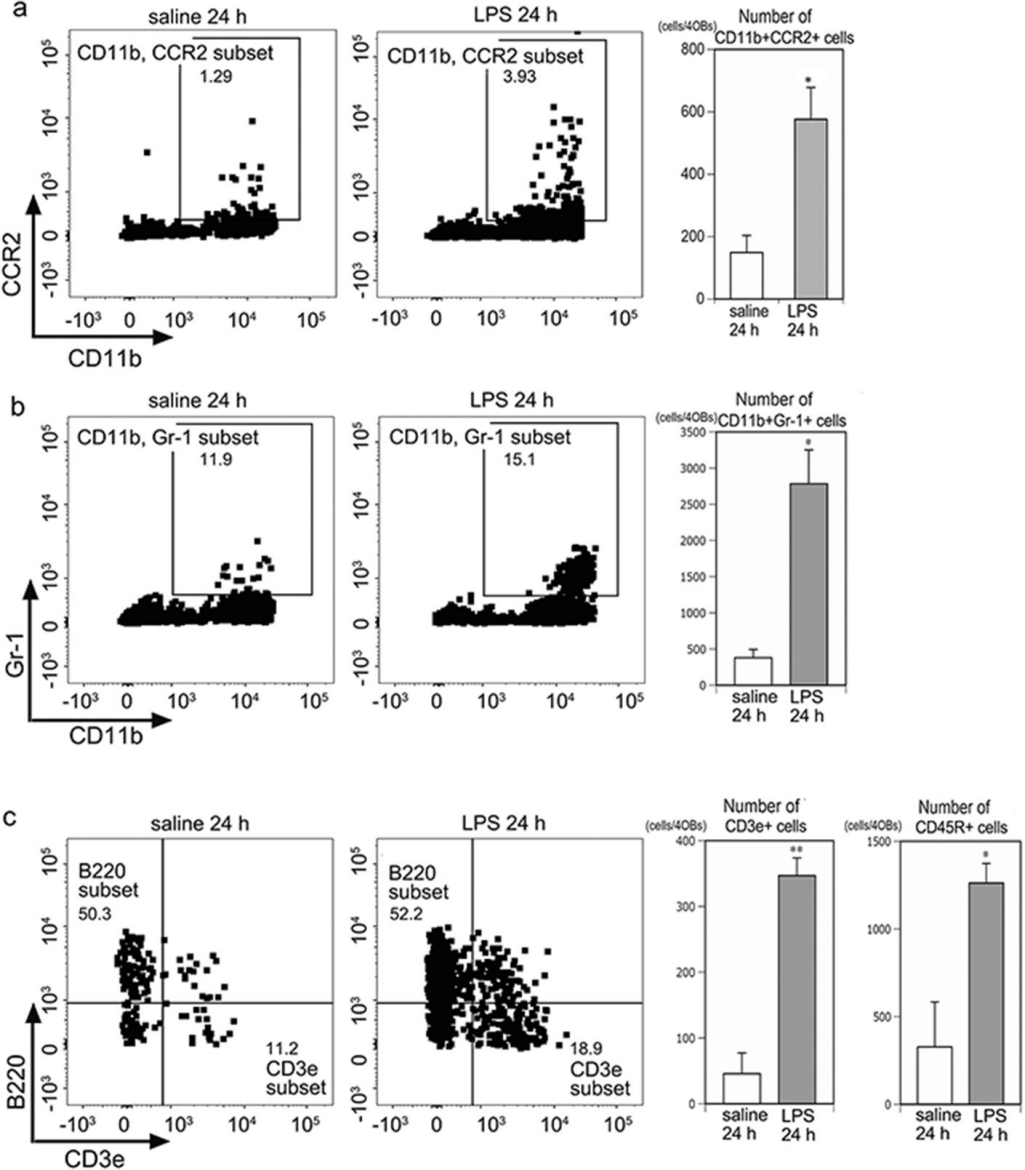
Flow cytometry. a Graphs showing the presence of CD11b + CCR2+ cells in the four OBs at 24 h post saline and 24 h post LPS. The number of CD11b + CCR2+ myeloid cells in the OB was significantly higher in the LPS24 h group than in the saline controls. *p < 0.05 compared with the saline controls, n = 3 experiments with two saline24 h mice and two LPS24 h mice per experiment. b Graphs showing the presence of CD11b + Gr-1+ cells in the four OBs at 24 h post saline and 24 h post LPS. The number of CD11b + Gr-1+ myeloid cells in the OB was significantly higher in the LPS24 h group than in the saline controls. *p < 0.05 compared with the saline controls. n = 4 experiments with two saline24 h mice and two LPS24 h mice per experiment. c Graphs showing the presence of B220+ or CD3e + cells in the four OBs at 24 h post saline and 24 h post LPS. The numbers of B220+ and CD3e + cells in the OB were both significantly higher in the LPS24 h group than in the saline controls. *p < 0.05, **p < 0.01 compared with the saline controls. n = 4 experiments with two saline24 h mice and two LPS24 h mice per experiment.

To confirm the histological results showing peripheral immune cell infiltration of the OB, we compared the number of leukocytes in the OBs using flow cytometry (Fig. 5). The number of the CD11b + CCR2+ subset was higher at 24 h post LPS than in the saline controls (Fig. 5a). The number of the CD11b + Gr-1+ subset was also higher in the OBs at 24 h post LPS than in the saline controls (Fig. 5b). Similarly, lymphocytes of the CD3e + or B220+ subset, were present in higher numbers in the OBs at 24 h post LPS than in the saline controls (Fig. 5c), indicating that peripheral immune cell numbers increased in the OB after intranasal LPS administration. Thus, both immunohistochemistry and flow cytometry revealed the infiltration of peripheral immune cells into the OB following intranasal LPS administration.

### 3.5. Expression of chemokines in the OB

**Fig. 6.**
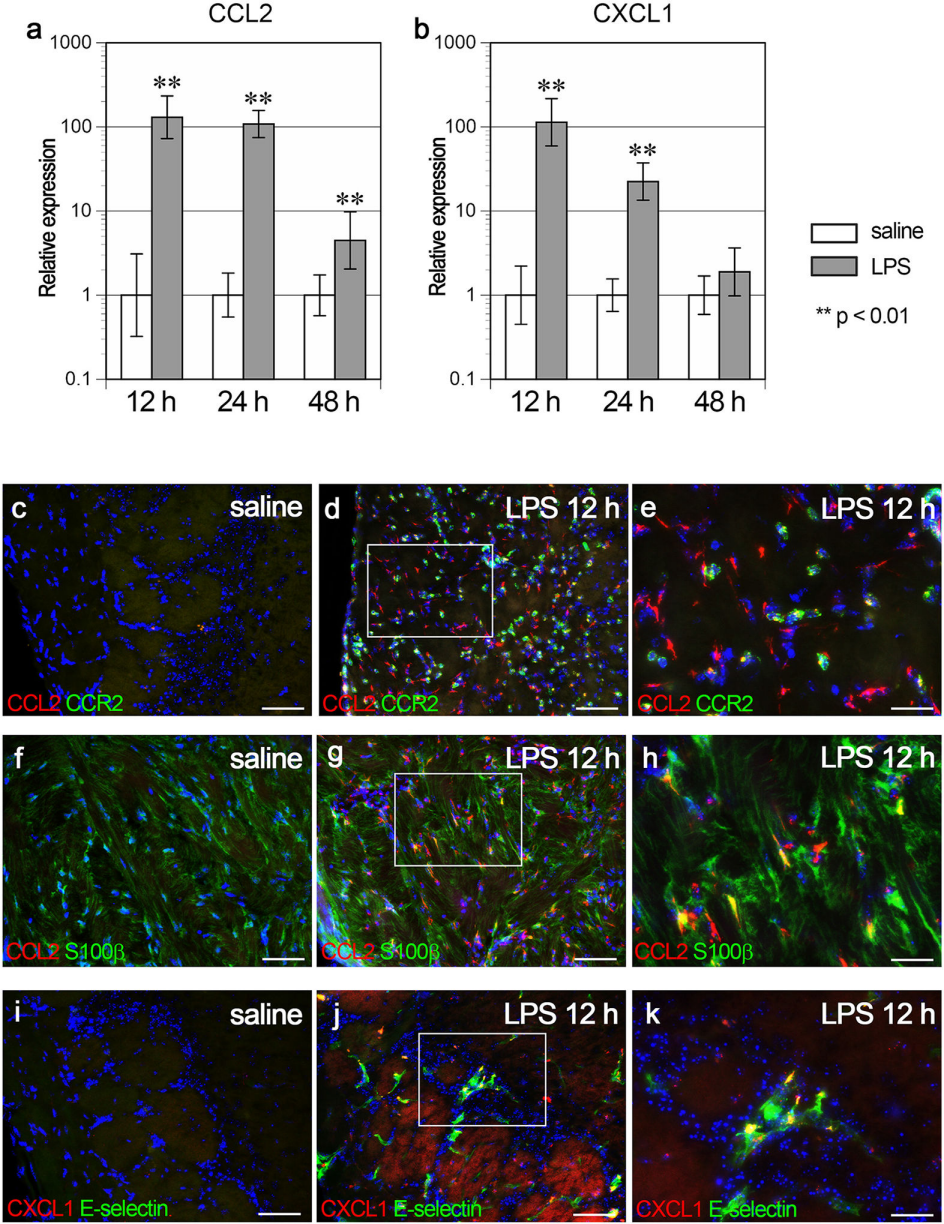
Chemokine expression in the OB. a, b Graphs showing the relative gene expression levels of CCL2 (a) and CXCL1 (b). Error bars are based on the SD of the ΔCT value. **p < 0.01, compared with the saline controls. The gene expression levels of CCL2 and CXCL1 were significantly higher at 12 h and 24 h post LPS than in the saline controls. n = 5 for each group with the exception of n = 4 for the saline12 h group. c-e, Immunofluorescence of CCL2 (red), CCR2 (green), and nuclei (DAPI, blue) in the OB of the saline control (c) and at 12 h post LPS (d and e). (e) is a magnified view of a framed box in (d). The higher magnification image confirms the co-localization. Scale bars, 50 μm in (c) and (d), and 20 μm in (e). f-h Immunofluorescence of CCL2 (red), S100β (green), and nuclei (DAPI, blue) in the OB of the saline control (f) and at 12 h post LPS (g and h). (h) is a magnified view of the framed box in (g). The higher magnification image confirms the co-localization. Scale bars, 50 μm in (f) and (g), and 20 μm in (h). i-k Immunofluorescence of CXCL1 (red), E-Selectin (green), and nuclei (DAPI, blue) in the OB of the saline control (i) and at 12 h post LPS (j) and (k). (k) is a magnified view of the framed box in (j). The higher magnification image confirms the co-localization. Scale bars, 50 μm in (i) and (j), and 20 μm in (k). Most CXCL1-immunopositive cells were also positive for E-Selectin at 12 h post LPS (k).

Since peripheral immune cells infiltrated the OB, we examined the expression levels of chemokines in the OB using real time RT-PCR. The expression of CCL2, a chemokine involved in monocyte recruitment, was 130-, 108-, and 4.5-fold higher at 12 h, 24 h and 48 h post LPS, respectively, than in the saline controls (p < 0.01 Fig. 6a). Double immunofluorescence staining revealed that CCL2 was not expressed in the saline control (Fig. 6c and f), but was expressed mainly by infiltrating CCR2-immunopositive cells (Fig. 6d and e) and S100β-immunopositive fibers in the ONL (Fig. 6g and h), which correspond to the olfactory ensheathing cells in the LPS12 h group. The expression of CXCL1, a chemokine involved in neutrophil recruitment, was 114- and 22.4-fold higher at 12 h and 24 h post LPS, respectively, than in the saline controls (p < 0.01 Fig. 6b). Double immunofluorescence staining revealed that CXCL1 was not expressed in the saline control (Fig. 6i), but was expressed by E-selectin-immunopositive endothelial cells in the LPS12 h group (Fig. 6j and k).

### 3.6. Activation of resident microglia

**Fig. 7.**
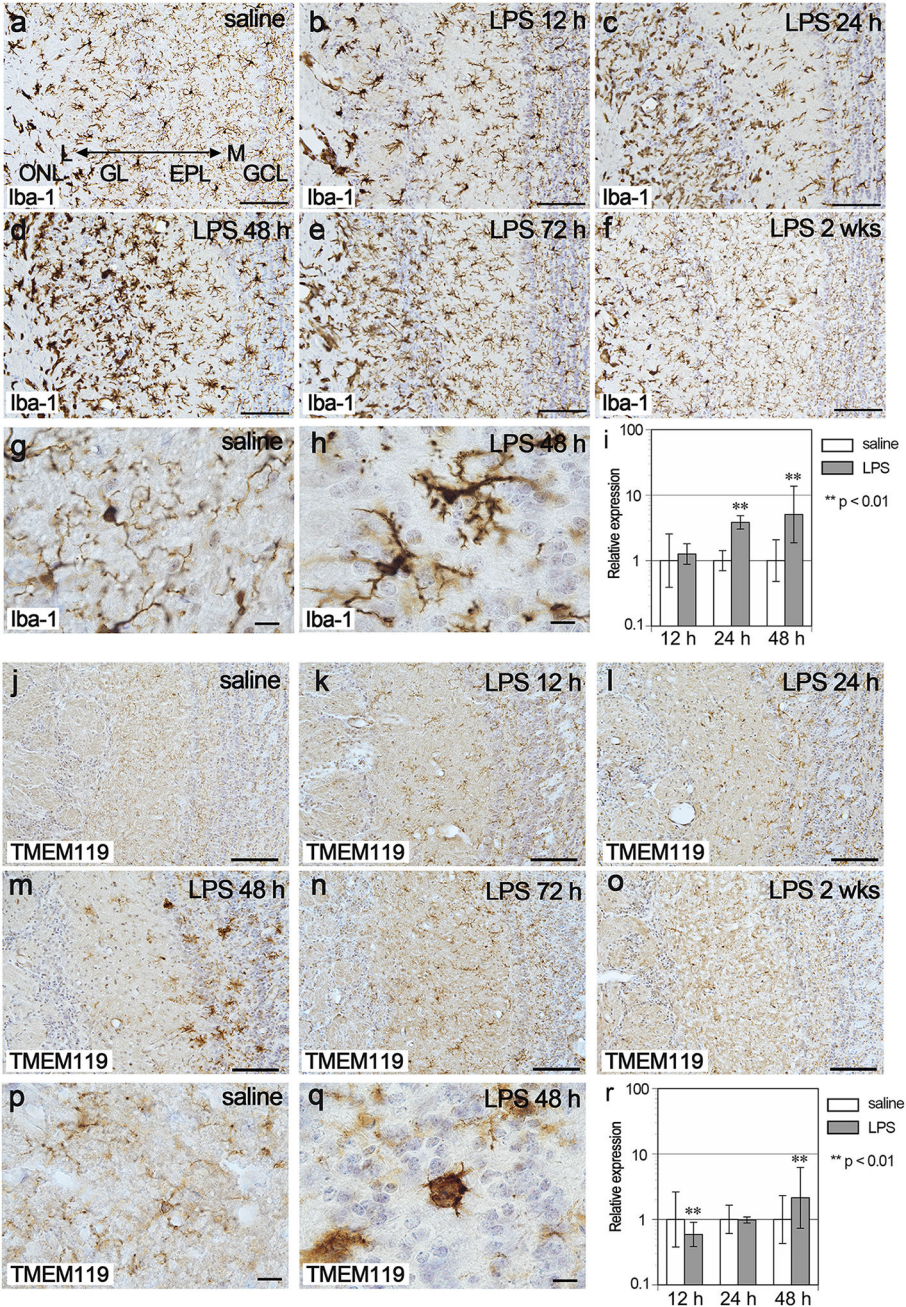
Activation of resident microglia in the OB. a-h Immunohistochemistry for Iba-1 in the lateral side of the OB in the saline (a, g), LPS12 h (b), LPS24 h (c), LPS48 h (d, h), LPS72 h (e), and LPS2 wks (f) groups. Scale bars: 100 μm (a-f) and 10 μm (g, h). Iba-1-immunopositive microglia were morphologically most activated at 48 h post LPS (d, h). i Graph showing the relative gene expression levels of Iba-1. Error bars are based on the SD of the ΔCT value. **p < 0.01, compared with the saline controls. The expression of Iba-1 was significantly higher at 24 h and 48 h post LPS. n = 5 for each group with the exception of n = 4 for the saline12 h group. j-q Immunohistochemistry for TMEM119 in the lateral side of the OB in the saline (j, p), LPS12 h (k), LPS24 h (l), LPS48 h (m, q), LPS72 h (n) and LPS2 wks (o) groups. Scale bars: 100 μm (j-o) and 10 μm (p, q). TMEM119-immunopositive microglia were morphologically most activated at 48 h post LPS (m, q). r Graph showing the relative gene expression levels of TMEM119. **p < 0.01, compared with the saline controls. The expression of TMEM119 was significantly higher at 48 h post LPS. n = 5 for each group with the exception of n = 4 for the saline12 h group.

Iba-1-immunopositive cells in the OB, corresponding to microglia, had small cell bodies with fine processes and were localized evenly in the brain tissue in the saline controls (Fig. 7a, g). After intranasal LPS administration, the cell bodies became larger and processes became thicker. Iba-1-immunopositive cells accumulated mainly on the lateral side of the OB (Fig. 7b-d). This morphological change of microglia peaked at 48 h post LPS (Fig. 7d, h) and returned to normal until 2 wks post LPS (Fig. 7e and f). In tandem with the morphological changes, the expression levels of Iba-1 in the OB were significantly 3.8- and 5.1-fold higher at 24 h and 48 h post LPS, respectively, than in the saline controls (Fig. 7i).

Iba-1 is not only expressed by microglia, but also by peripheral macrophages. To identify the resident endogenous microglia, we used an antibody against TMEM119, which is specifically expressed by microglia but not by macrophages (Bennett et al., 2016). In the saline controls, microglial processes were weakly immunopositive for TMEM119 (Fig. 7j and p). They became more strongly immunopositive at 12 h and 24 h post LPS (Fig. 7k, l). At 48 h post LPS, the cells that strongly expressed TMEM119 increased in number in the lateral sides of the dEPL and GCL and had round cell bodies with short and thick processes (Fig. 7m and q). At 72 h, these cells disappeared, and the expression levels returned to normal at 2 wks post LPS (Fig. 7n and o). The expression levels of TMEM119 in the OB increased slightly by 2.1-fold at 48 h post LPS, whereas its expression decreased at 12 h post LPS (Fig. 7r). At 48 h post LPS, resident microglia proliferated with Ki-67 expression (Supplementary Fig. 2).

### 3.7. Expression of cytokines in the OB

**Fig. 8.**
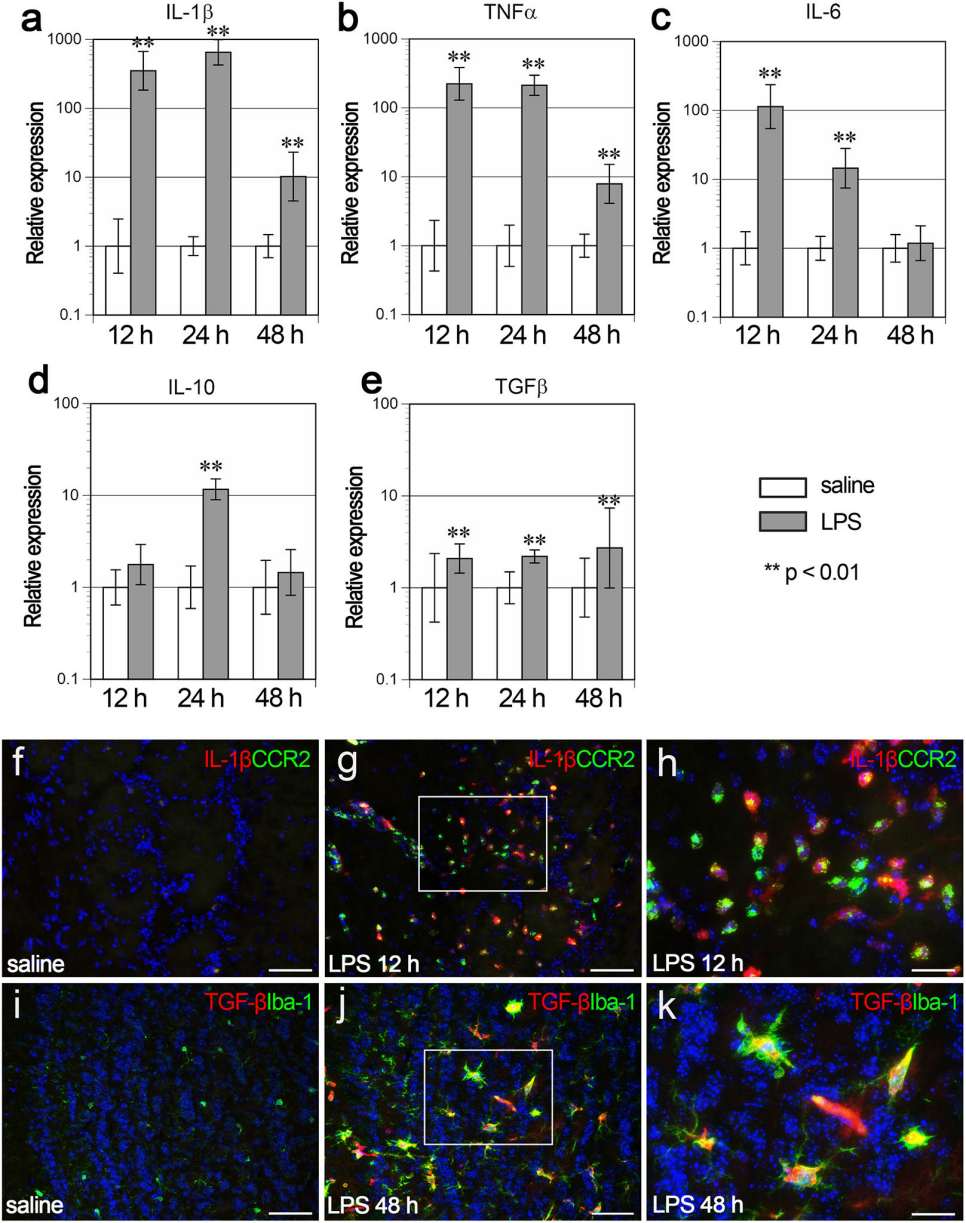
Expression of cytokine in the OB. a-e Graphs showing the relative gene expression levels of IL-1β (a), TNFα (b), IL-6 (c), IL-10 (d), and TGFβ (e). Error bars are based on the SD of the ΔCT value. **p < 0.01, compared with the saline controls. The gene expression levels of IL-1β and TNFα were significantly higher at 12 h, 24 h, and 48 h post LPS than in the saline controls, respectively (a, b). The gene expression levels of IL-6 were significantly higher at 12 h and 24 h post LPS than in the saline controls, respectively (c). The gene expression levels of IL-10 were significantly higher at 24 h post LPS than in the saline control (d). The gene expression levels of TGFβ were significantly higher at 12 h, 24 h and 48 h post LPS than in the saline controls (e). n = 5 for each group with the exception of n = 4 for the saline12 h group. f-h Immunofluorescence of IL-1β (red), CCR2 (green), and nuclei (DAPI, blue) in the OB of the saline control (f) and at 12 h post LPS (g and h). IL-1β was expressed mainly by CCR2-immunopositive cells at 12 h post LPS (g and h). (h) is a magnified view of the framed box in (g). The higher magnification image confirms the co-localization. Scale bars, 50 μm in (f) and (g) and 20 μm in (h). i-k Immunofluorescence of TGFβ (red), Iba-1 (green), and nuclei (DAPI, blue) in the OB of the saline control (i) and at 48 h post LPS (j and k). TGFβ was expressed mainly by microglia in the EPL and GCL of the OB at 48 h post LPS (j and k). (k) is a magnified view of the frame box in (j). The higher magnification image confirms the co-localization. Scale bars, 50 μm in (i) and (j) and 20 μm in (k).

The expression of pro-inflammatory cytokines (IL-1β, TNFα, and IL-6) and anti-inflammatory cytokines (IL-10 and TGFβ) was examined in the OB using real time RT-PCR. The expression of IL-1β was 351-, 650-, and 10.2-fold higher at 12 h, 24 h, and 48 h post LPS than in the saline controls, respectively (Fig. 8a). The expression of TNFα was 223-, 213-, and 7.9-fold higher at 12 h, 24 h and 48 h post LPS than in the saline controls, respectively (Fig. 8b). The expression of IL-6 was 114- and 14.5-fold higher at 12 h and 24 h post LPS than in the saline controls, respectively, but returned to a normal level at 48 h post LPS (Fig. 8c). The expression of IL-10 was 11.7-fold higher at 24 h post LPS than in the saline controls (Fig. 8d), and the expression of TGFβ was 2.1-, 2.2-, and 2.7-fold higher at 12 h, 24 h, and 48 h post LPS than in the saline controls, respectively, indicating that IL-10 expression was transiently elevated and TGFβ was slightly but significantly increased in the OB after intranasal LPS administration (Fig. 8e).

Double immunofluorescence revealed that IL-1β was not expressed in the saline control (Fig. 8f), but expressed mainly by CCR2-immunopositive cells in the ONL and GL at 12 h post LPS (Fig. 8g and h). A few IL-1β-immunopositive cells were double positive for TMEM119 in the EPL. TGFβ was not expressed in the saline control (Fig. 8i), but expressed by Iba-1-immunopositive cells in the EPL and GCL at 48 h post LPS (Fig. 8j and k), indicating that resident microglia released TGFβ.

### 3.8. Changes in the meningeal immunity

**Fig. 9.**
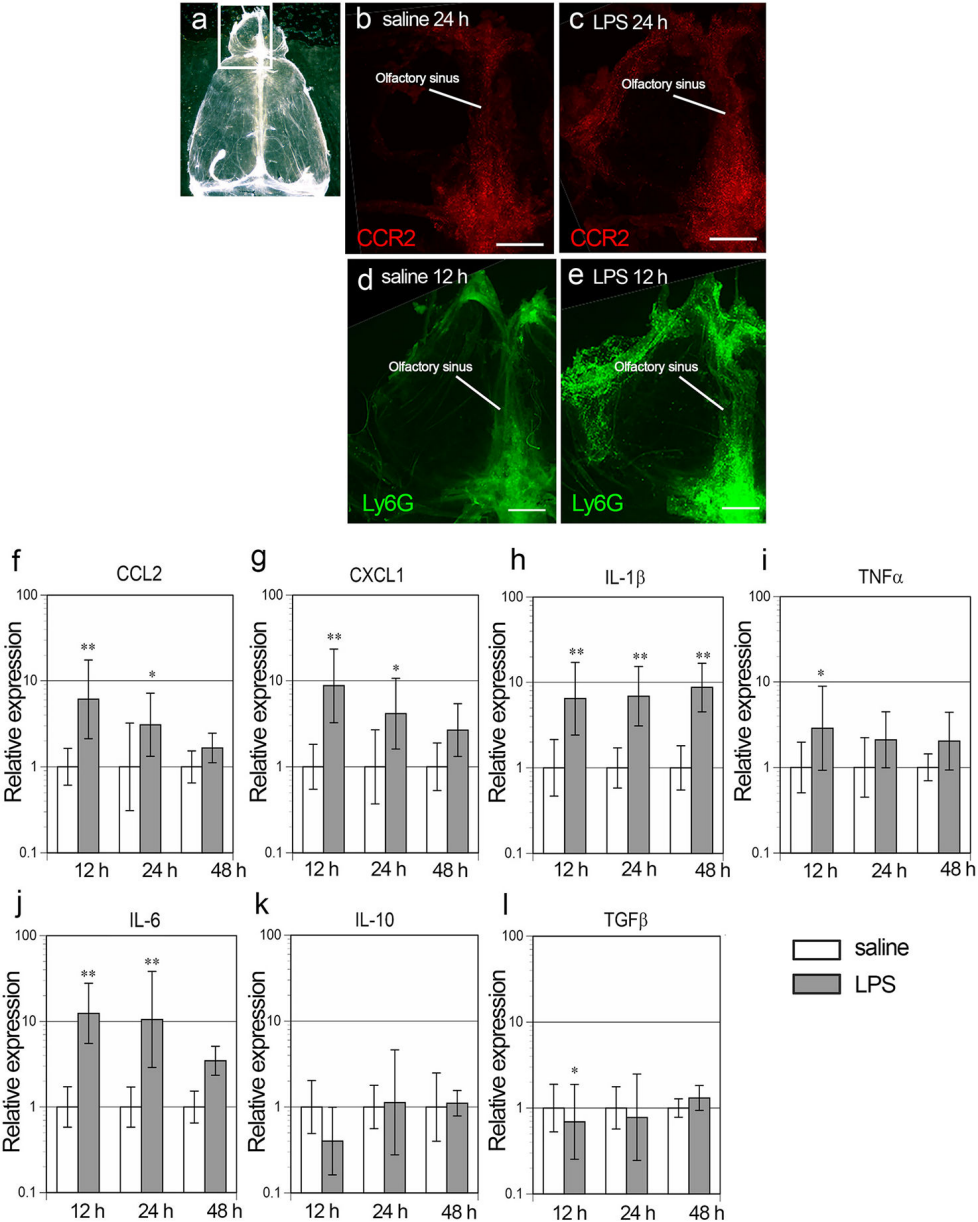
Activation of the meningeal immunity. a Whole mount meninges. A rectangle indicates the area shown in b-e. b, c Immunofluorescence of CCR2 at 24 h post saline (b) and LPS (c). d, e Immunofluorescence of Ly6G at 12 h post saline (d) and 12 h LPS (e). More CCR2- and Ly6G-immunopositive cells accumulated in the olfactory sinus post LPS. Scale bars, 500 μm. f-l Graphs showing the relative gene expression levels of CCL2 (f), CXCL1 (g), IL-1β (h), TNFα (i), IL-6 (j), IL-10 (k), and TGFβ (l). Error bars are based on the SD of the ΔCT value. *p < 0.05, **p < 0.01, compared with the saline controls. The gene expression levels of CCL2, CXCL1, IL-1β, TNFα and IL-6 were significantly higher in the LPS-treated mice than in the saline controls, while those of IL-10 or TGFβ were not. n = 5 for each group with the exception of n = 4 for the saline12 h group.

Next, we examined changes in the meningeal immunity after intranasal LPS administration. Whole mount tissue immunostaining showed that CCR2-immunopositive cells were present within the olfactory sinus in the meninges of the saline controls, and that their number was particularly high at 24 h post LPS (Fig 9a-c). Ly6G-immunopositive cells were not seen in the meninges of the saline control, but accumulated in the olfactory sinus in the meninges at 12 h post LPS (Fig 9d, e).

We compared the expression levels of cytokines and chemokines in the meninges after intranasal administration of saline or LPS using real time RT-PCR. The expression of CCL2 was 6.1- and 3.1-fold higher at 12 h and 24 h post LPS, respectively, than in the saline controls (Fig. 9f). Similarly, the expression of CXCL1 was 8.8- and 4.1-fold higher at 12 h and 24 h post LPS, respectively (Fig. 9g).

The expression of pro-inflammatory cytokines increased after intranasal LPS administration. IL-1β was 6.5-, 6.9-, and 8.7-fold higher at 12 h, 24 h and 48 h post LPS, respectively, than in the saline controls (Fig. 9h). TNFα was significantly 2.9-fold higher at 12 h than in the saline controls, but its level did not change at 24 h or 48 h post LPS (Fig 9i). IL-6 was significantly 12.4- and 10.5-fold higher at 12 h and 24 h post LPS, respectively, than in the saline controls (Fig 9i and j). The expression of anti-inflammatory cytokines, such as IL-10 or TGFβ, did not increase after intranasal LPS administration, rather TGFβ significantly decreased at 12 h post LPS (Fig. 9k and l).

## 4. Discussion

In the present study, we found that intranasal LPS administration caused transient infiltration of peripheral immune cells and an increase in the levels of inflammatory molecules in the OB within 12 h, and that this was associated with the activation of olfactory nerve fibers (a component of the neural route) and meningeal immunity (a component of the meningeal route). Resident microglia in the OB were activated and produced anti-inflammatory cytokines within 24 h post LPS, which could play a role in recovery from inflammation.

### 4.1. The infiltration of peripheral immune cells to the brain

Tight junction of the brain blood vessels together with astrocytes form the major elements of the blood brain barrier (BBB) that prevents blood-derived cells and molecules from entering the brain parenchyma freely. However, immune cells can enter the brain parenchyma under some pathological and psychiatric conditions. For instance, following ischemic stroke, reactive oxygen species and pro-inflammatory factors upregulate cell adhesion molecules on leukocytes and endothelial cells, which allows blood-derived leukocytes to infiltrate the ischemic area (Jayaraj et al., 2019). Chronic alcohol consumption promotes the recruitment of peripheral macrophages into the CNS and microglia alteration through the CCR2/5 axis (Lowe et al., 2020). In multiple sclerosis and experimental autoimmune encephalomyelitis (EAE), infiltrating monocytes accumulate in the nodes of Ranvier and initiate demyelination (Yamasaki et al., 2014). Neutrophils and CCR2+ inflammatory monocytes are recruited to the brains of both patients and mice suffering from sepsis-associated encephalopathy via increases in chemokine expression and subtle microglial activation, causing persistent cognitive impairment (Andonegui et al., 2018). In addition, monocyte recruitment to the brain via CCL2 and CX3CL1 signaling in response to repeated social stress has been reported in rodents (Wohleb et al., 2013; Wohleb and Delpech, 2017). Peripheral monocytes and resident microglia are activated in the brain by repeated stress exposure, leading to the development of anxiety- and depressive-like behaviors (Wohleb et al., 2013, Wohleb and Delpech, 2017). The present study has allowed us to add another effector to this list, i.e. nasal inflammation, which causes the infiltration of CCR2-immunopositive cells, Ly6G-immunopositive cells, and lymphocytes to the OB transiently and in a very short time. In addition, nasal inflammation caused similar associated events including upregulation of cell adhesion molecules, microglial activation, and elevation of chemokine and cytokine expression with infiltration of peripheral immune cells into the brain. Thus, these may be the common initial events that occur in the brain under some pathological and psychiatric conditions.

### 4.2. Routes from nose to brain

Olfactory ensheathing cells wrap the axon bundles of olfactory sensory neurons from the lamina propria in the olfactory mucosa to the glomeruli in the OB through the cribriform plate. Among the olfactory nerve fiber bundles, a few monocytes are present, even under normal conditions (Smithson and Kawaja, 2010). Olfactory ensheathing cells and monocytes are known to express toll-like receptor 4 (TLR4), which is a receptor for LPS (Vincent et al., 2007; Leung et al., 2008). Thus, when LPS is administered into the nasal cavity, olfactory ensheathing cells and monocytes become rapidly activated. Indeed, we found that S100β-immunopositive fibers in the ONL, which may correspond to olfactory ensheathing cells (Rawji et al., 2013), were double positive for CCL2, and that CCR2-immunopositive cells were distributed along the olfactory nerve fibers in the ONL. This suggests that CCR2-immunopositive cells could reach the OB via CCL2-expressing olfactory ensheathing cells. Thus, the olfactory nerve fibers with ensheathing cells could act as a route for their migration to the OB.

In addition, we observed the activation of meningeal immunity. The dura mater contains a rich diversity of immune cells particularly around the dural sinuses in physiological conditions (Alves de Lima et al., 2020; Rustenhoven et al., 2021). We found that, after intranasal LPS administration, the numbers of CCR2- and Ly6G-immunopositive cells increased in the olfactory sinus and pro-inflammatory cytokines were markedly upregulated in the meninges. When trypan blue was administered to the nasal cavity, no leakage of blue dye was observed in the meninges, indicating that LPS had not been transported into the meninges. Thus, meningeal cells in the dura mater may be activated not by LPS directly. Instead, activated immune cells, olfactory ensheathing cells, and cells of the OE may release inflammatory molecules that propagate to the meningeal space through the olfactory nerve fibers to stimulate meningeal cells. Cytokines released by cells in the dura mater likely access the cerebrospinal fluid and furthermore the brain parenchyma to induce neuroinflammation, although it still remains unknown how dural cytokines penetrate the arachnoid mater to reach the cerebrospinal fluid (Alves de Lima et al., 2020). Moreover, increased levels of cytokines in the meninges could induce the activation of blood vessels running in the subarachnoid space and penetrating the brain parenchyma. This could result in the upregulation of the expression of cell adhesion molecules in the endothelial cells (Supplementary Fig. 3), which might transiently increase the permeability of the BBB and contribute to the infiltration of immune cells into the OB (Abbott, 2000).

### 4.3. Resident microglia vs infiltrating CCR2-immunopositive cells

Endogenous resident microglia and infiltrating exogenous CCR2-immunopositive cells are thought to have different features and to contribute differently to the pathophysiology of diseases. In an EAE model, infiltrating monocytes are activated and release pro-inflammatory factors, such as IL-1β and iNOS, which contribute to the onset of demyelination (Yamasaki et al., 2014). By contrast, resident microglia are activated and produce anti-inflammatory factors, such as IGF-1 and TGFβ, in the middle and late phases of EAE, which promotes recovery from inflammation (Yamasaki et al., 2014). In the present study, we distinguished endogenous resident microglia and exogenous myeloid cells by using the anti-TMEM119 antibody to stain microglia (Bennett et al., 2016) and the anti-CCR2 antibody to stain a subset of peripheral myeloid cells, which revealed clear differences in terms of their distribution, morphology, the way their number increases, and their potential function. In the OB, TMEM119-immunopositive resident microglia had ramified processes, and were distributed in the EPL and GCL, but not in the ONL or GL in the saline controls. Since Iba-1-immunopositive cells were found in all layers of the OB, including the ONL and GL, Iba-1-immunopositive cells may contain cells different from resident microglia. After intranasal LPS administration, CCR2-immunopositive myeloid cells, which had no ramified processes, mainly infiltrated the ONL and GL and extended to the sEPL, but were not conspicuous in the deeper layers of the OB, namely the dEPL and GCL. At 48 h after intranasal LPS administration, TMEM119-immunopositive resident microglia proliferated as evidenced by the increased Ki-67 expression associated with the upregulation of TMEM119 expression. By contrast, the number of CCR2-immunopositive myeloid cells increased via infiltration. In addition, infiltrating CCR2-immunopositive cells expressed pro-inflammatory factors, such as IL-1β, and CCL2, at 12–24 h after intranasal LPS administration, while resident microglia in the EPL and GCL began to express anti-inflammatory cytokines, such as TGFβ, at 48 h. This indicates that exogenous myeloid cells made the brain microenvironment pro-inflammatory early after LPS administration, and that resident microglia contributed to the return to normal.

According to previous reports, exogenous monocytes do not turn into resident microglia (Bennett et al., 2016), but it is possible that exogenous monocytes change their phenotype after infiltrating the brain. On Day 3 after stroke in mice, monocyte-derived macrophages infiltrate the stroke-injured brain from the circulation and most of them exhibit a bias toward a pro-inflammatory phenotype (Wattananit et al., 2016). However, during the subsequent 2 wks, they shift to an anti-inflammatory phenotype and contribute to long-term functional recovery after stroke (Wattananit et al., 2016). Thus, following the early period of nasal inflammation, infiltrating CCR2-immunopositive cells may switch to an anti-inflammatory phenotype and contribute to olfactory bulb recovery together with resident microglia.

### 4.4. Potential association of infiltrating immune cells with OB atrophy and brain dysfunction

In our previous study, we found that the OB is significantly atrophied after 10 wks of repeated intranasal LPS administration and that tufted cells are more damaged than mitral cells (Hasegawa-Ishii et al., 2019; Hasegawa-Ishii et al., 2020). The reason why tufted cells are more vulnerable to nasal inflammation remains unknown. In the present study, we found that peripheral immune cells transiently infiltrated the ONL, GL, and sEPL. Given that tufted cells extend their secondary dendrites to the sEPL and mitral cells to the dEPL, tufted cells and their dendrites may be exposed to infiltrating immune cells at the early period of nasal inflammation. Activated immune cells can release pro-inflammatory cytokines and some neurotoxic molecules, such as NO and glutamate (Spranger et al., 1996; Kobayashi, 2010), which could cause shrinkage of the spines and dendrites of neurons (Hasegawa et al., 2004). If these small damages accumulate during chronic nasal inflammation, the layer of tufted cells could shrink, and cause whole OB atrophy. A smaller OB may decrease odor input, leading to olfactory dysfunction. Considering that patients with depression and anxiety have a reduced OB volume and olfactory dysfunction (Negoias et al., 2010; Asal et al., 2018; Rottstadt et al., 2018; Rottstaedt et al., 2018), our findings may contribute to understanding the mechanisms underlying the connection between nasal inflammation and psychiatric disorders.

### 4.5. Limitation

First, we used LPS solution to trigger nasal inflammation in the mouse nostril, which could be considered artificial, because natural agents in the environment, such as bacteria and viruses, enter the nostril by inhalation. Second, we focused on innate immune cells such as CCR2-and Ly6G-immunopositive cells since these cells initially responded to nasal inflammation. However, T lymphocytes, for example, could also play roles in both initiation and termination of inflammation. Particularly T lymphocytes in the meninges could contribute to changes in the brain cytokine microenvironment (Derecki et al., 2010; Laumet et al., 2018). Third, we analyzed immune cell infiltration by immunohisto-chemistry, which provided useful information on the time course and critical period of inflammation; however, it does not provide information on the movements of live cells.

## 5. Conclusion

In the early period, an acute episode of nasal inflammation causes the transient infiltration of the OB by peripheral immune cells, which is associated with the activation of olfactory nerve fibers and meningeal immunity, resulting in the development of a pro-inflammatory status. After this period, resident microglia become activated and release anti-inflammatory cytokines. This could be the initial events that lead to OB atrophy.

## Supplementary Material

Supplementary data

## Figures and Tables

**Table 1:** List of primary antibodies.

Against	Host	Source	Dilution	Cat. number	Antigen retrieval
CCR2	rabbit	Abcam	1:250	ab273050	〇 (T)
Ly6G	rat	AdipoGen	1:200	AG-20B-0043PF	ß
CD3e	rabbit	ThermoFisher Scientific	1:150	MA1–90582	〇 (C)
CD45R	rat	Abcam	1:200	ab64100	〇 (C)
Iba-1	rabbit	Abcam	1:2000	ab178846	〇 (T)
Iba-1	goat	Abcam	1:300	ab107159	〇 (T)
TMEM119	rabbit	Abcam	1:300	ab209064	〇 (C)
IL-1β	goat	Abcam	1:200	ab9722	×
CCL2	goat	R&D Systems	1:200	AF-479-NA	×
CXCL1	goat	R&D Systems	1:100	AF-453-NA	×
E-Selectin	chicken	R&D Systems	1:100	AF575	〇 (T)
VCAM-1	rabbit	Abcam	1:500	ab134047	〇 (T)
ICAM-1	goat	R&D Systems	1:100	AF796	〇 (T)
TGFβ	rabbit	Abcam	1:500	ab215715	〇 (T)
S100β	rabbit	Abcam	1:500	ab52642	〇 (C)
Ki-67	rabbit	Abcam	1:200	ab16667	〇 (T)
Ki-67	rat	Invitrogen	1:1000	14–5698-82	〇 (T)

Information on all primary antibodies used for immunostaining is listed, including the name, host, source, dilution, catalog number, and necessity of antigen retrieval.

(T), Tris-EDTA buffer; (C), Citrate buffer.

**Table 2:** List of TaqMan probes.

Target	Catalog number
GAPDH	Mm9999915_g1
Hprt	Mm03024075_m1
IL-6	Mm00446190_m1
Tnf	Mm00443258_m1
IL-1b	Mm01336189_m1
CCL2	Mm00441242_m1
CXCL1	Mm04207460_m1
IL-10	Mm01288386_m1
Tgfb	Mm01178820_m1
TMEM119	Mm00525305_m1
Aif1	Mm00479862_g1

Information on all probes used in this study is listed.

## Abbreviations:

OE: olfactory epithelium
CRS: chronic rhinosinusitis
OB: olfactory bulb
LPS: lipopolysaccharide
EPL: external plexiform layer
ONL: olfactory nerve layer
GL: glomerular layer
sEPL: superficial EPL
dEPL: deep EPL
IL-1β: interleukin-1β
TNFα: tumor necrosis factor α
TGFβ: transforming growth factor β
TLR: toll like receptor
BBB: blood brain barrier
EAE: experimental autoimmune encephalomyelitis
iNOS: inducible nitric oxide synthase
IGF-1: insulin-like growth factor 1
NO: nitric oxide
h: hour
wks: weeks
