# Supplementary material for: Infiltration of peripheral immune cells into the olfactory bulb in a mouse model of acute nasal inflammation
Source: J Neuroimmunol. Author manuscript; Available in PMC 2023 Feb 7. (PMC9903215; doi:10.1016/j.jneuroim.2022.577897)
Supplement: Supplementary data [file NIHMS1866963-supplement-Supplementary_data.pdf]

 Click here to access/download;Graphical Abstract;Graphic abstract.tif

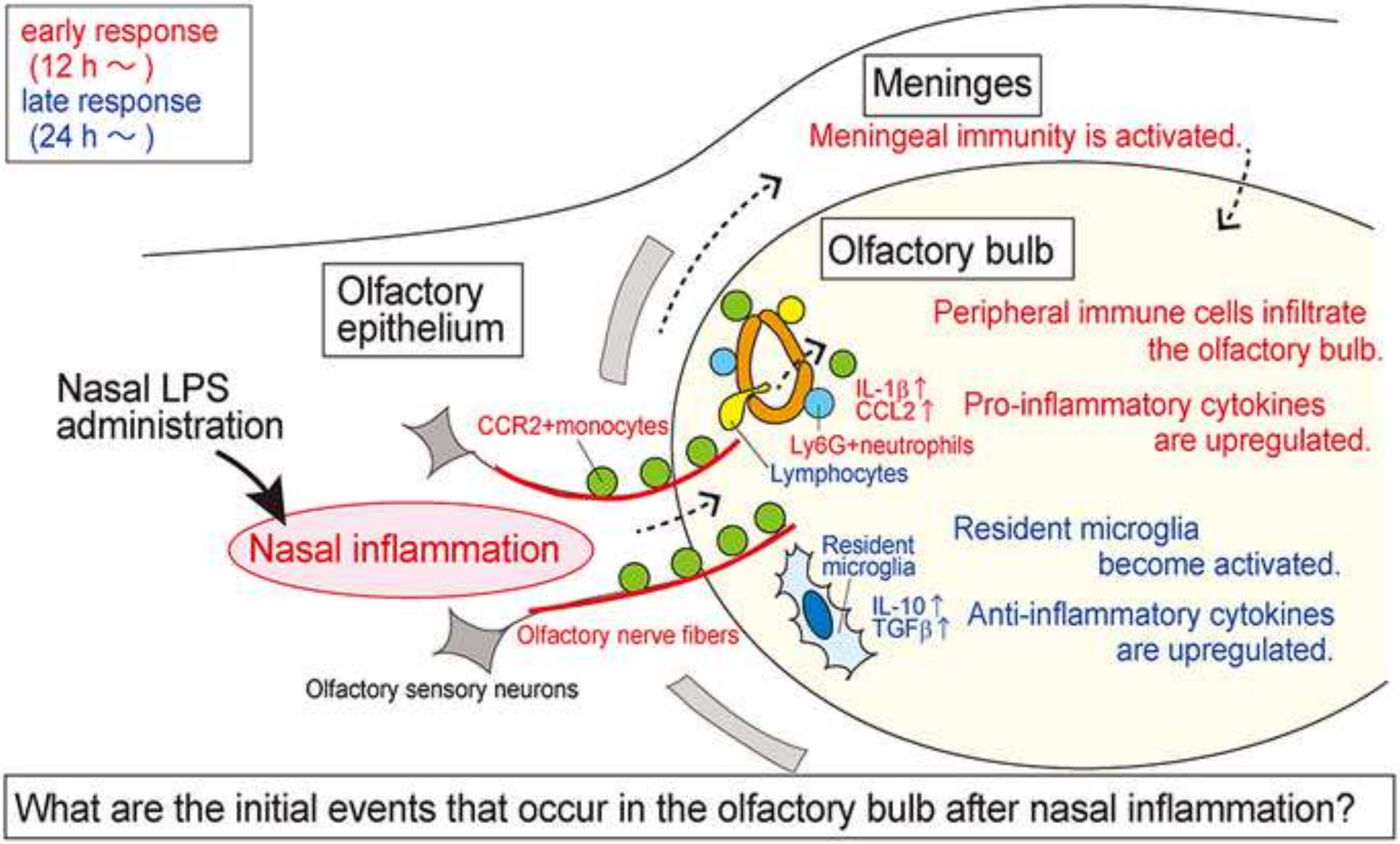

Manuscript File Click here to view linked References

Infiltration of peripheral immune cells into the olfactory bulb in a mouse model of acute nasal inflammation

Authors:

Hinami Asano[1], Sanae Hasegawa-Ishii[1]*, Ken Arae[2], Aki Obara[3], Geoffroy Laumet[4], Robert Dantzer[5] and Atsuyoshi Shimada[1]

Institutional address:

[1] Pathology Research Team, Faculty of Health Sciences, Kyorin University, 5-4-1 Shimorenjaku, Mitaka-shi, Tokyo 181-8612, Japan

[2] Department of Immunology, Faculty of Health Sciences, Kyorin University 5-4-1 Shimorenjaku, Mitaka-shi, Tokyo 181-8612, Japan

[3] Department of Analytical Chemistry, Faculty of Health Sciences, Kyorin University, 5-4-1 Shimorenjaku, Mitaka-shi, Tokyo 181-8612, Japan

[4] Department of Physiology, Michigan State University 766 Service Rd, East Lansing, MI 48824, USA

[5] Department of Symptom Research, Division of Internal Medicine, The University of Texas MD Anderson Cancer Center, 1515 Holcombe, Blvd. Houston, TX 77030, USA

Corresponding Author:

Sanae Hasegawa-Ishii

E-mail address: sanae_ishii@ks.kyorin-u.ac.jp

Address: Pathology Research Team, Faculty of Health Sciences, Kyorin University,

5-4-1 Shimorenjaku, Mitaka-shi, Tokyo 181-8612, Japan

Tel: +81-422-47-8000 (ex. 1506)

Competing interests

All authors declare that they have no competing interests.

Table 1. List of primary antibodies. Information on all primary antibodies used for immunostaining is listed, including the name, host, source, dilution, catalog number, and necessity of antigen retrieval.

Table 2. List of TaqMan probes. Information of all probes used in this study is listed.

Supplementary Figure 1. Engulfment of Ly6G-immunopositive debris by microglia. Immunofluorescence of Iba-1 (red), Ly6G (green), and nuclei (DAPI, blue) in the OB at 48 h post LPS. Scale bar, 50 μm. Ly6G-immunopositive debris without nuclei were engulfed by Iba-1-immunopositive microglia at 48 h post LPS.

Supplementary Figure 2. Proliferation of microglia in the OB

a-f Immunohistochemistry of Ki-67 in the lateral side of the OB in the saline control (**a**) and at 12 h (**b**), 24 h (**c**), 48 h (**d**), 72 h (**e**) and 2 wks (**f**) post LPS. Scale bars: 100 μm. **g** Immunofluorescence of Ki-67 (red), Iba-1 (green), and nuclei (DAPI, bule) at 48 h post LPS. Scale bars, 100 μm. Ki-67-immunopositive cells were double positive for TMEM119 at 48 h post LPS.

Supplementary Figure 3. Expression of cell adhesion molecules on endothelial cells

a, b Immunofluorescence of E-Selectin (green) and nuclei (DAPI, blue) in the OB in the saline control and at 12 h post LPS. **c, d** Immunofluorescence of VCAM-1 (red) and nuclei (DAPI, blue) in the OB in the saline control and at 12 h post LPS. **e, f** Immunofluorescence of ICAM-1 (red) and nuclei (DAPI, blue) in the OB in the saline control and at 12 h post LPS. Scale bars, 100 μm. The expression of cell adhesion molecules was elevated at 12 h

post LPS.

Author Agreement

All authors have seen and approved the final version of the manuscript. They warrant that the article is the authors' original work and has not received prior publication and is not under consideration for publication elsewhere.

Infiltration of peripheral immune cells into the olfactory bulb in a mouse model of acute nasal inflammation

Authors:

Hinami Asano[1], Sanae Hasegawa-Ishii[1]*, Ken Arae[2], Aki Obara[3], Geoffroy Laumet[4], Robert Dantzer[5] and Atsuyoshi Shimada[1]

Institutional address:

[1] Pathology Research Team, Faculty of Health Sciences, Kyorin University,
5-4-1 Shimorenjaku, Mitaka-shi, Tokyo 181-8612, Japan

[2] Department of Immunology, Faculty of Health Sciences, Kyorin University
5-4-1 Shimorenjaku, Mitaka-shi, Tokyo 181-8612, Japan

[3] Department of Analytical Chemistry, Faculty of Health Sciences, Kyorin University,
5-4-1 Shimorenjaku, Mitaka-shi, Tokyo 181-8612, Japan

[4] Department of Physiology, Michigan State University
766 Service Rd, East Lansing, MI 48824, USA

[5] Department of Symptom Research, Division of Internal Medicine, The University of Texas
MD Anderson Cancer Center, 1515 Holcombe, Blvd. Houston, TX 77030, USA

Corresponding Author:

Sanae Hasegawa-Ishii

E-mail address: sanae_ishii@ks.kyorin-u.ac.jp

Address: Pathology Research Team, Faculty of Health Sciences, Kyorin University,

5-4-1 Shimorenjaku, Mitaka-shi, Tokyo 181-8612, Japan

Tel: +81-422-47-8000 (ex. 1506)

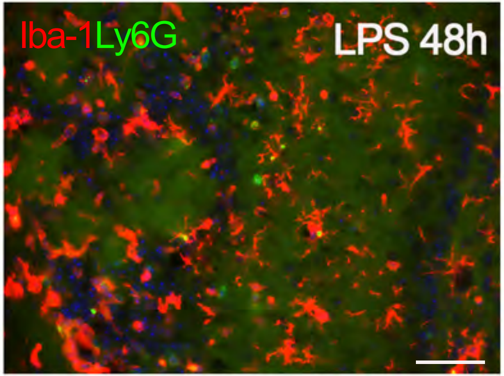
Iba-1 Ly6G
LPS 48h

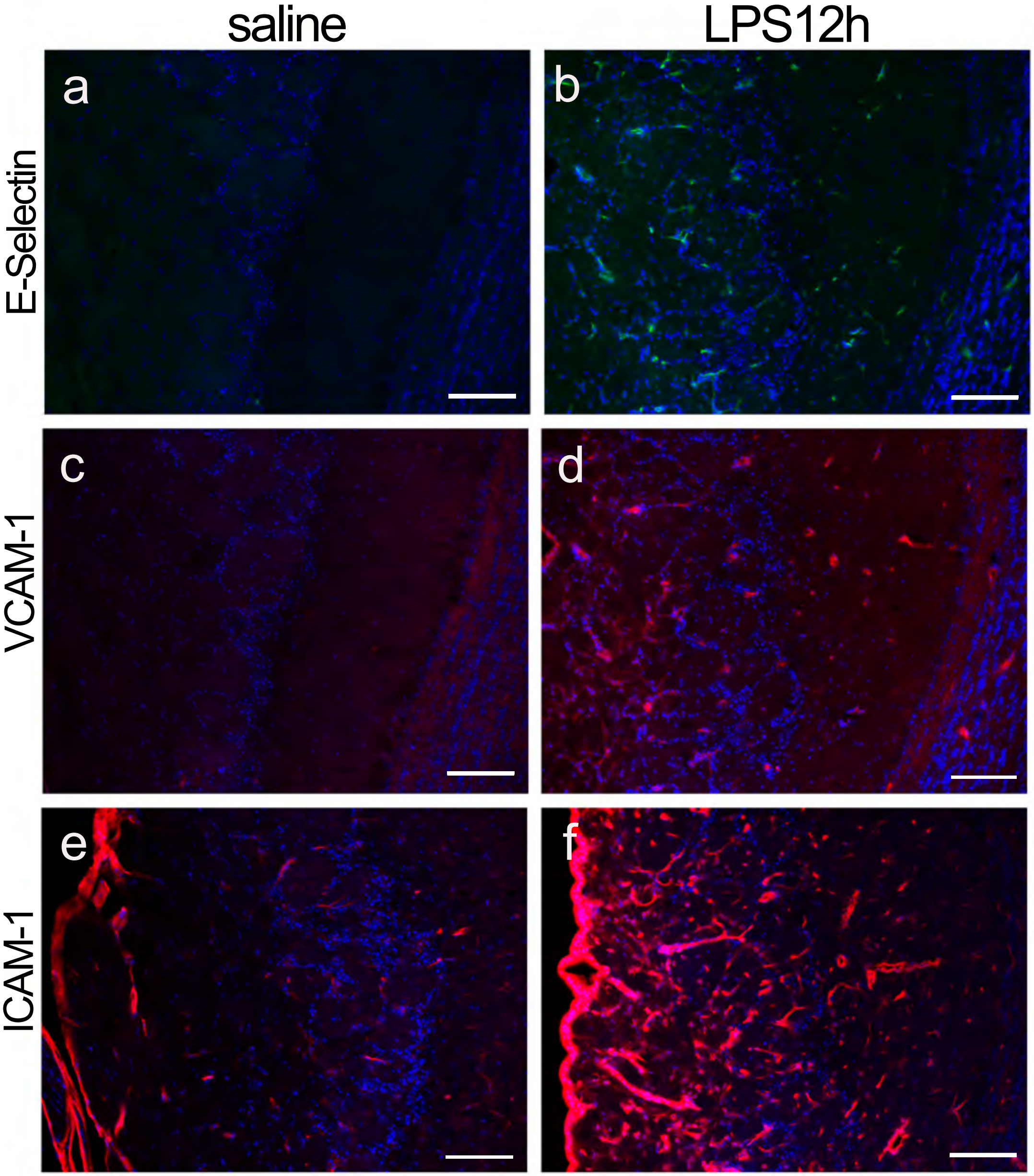
saline
LPS12h
E-Selectin
VCAM-1
ICAM-1
a
b
c
d
e
f

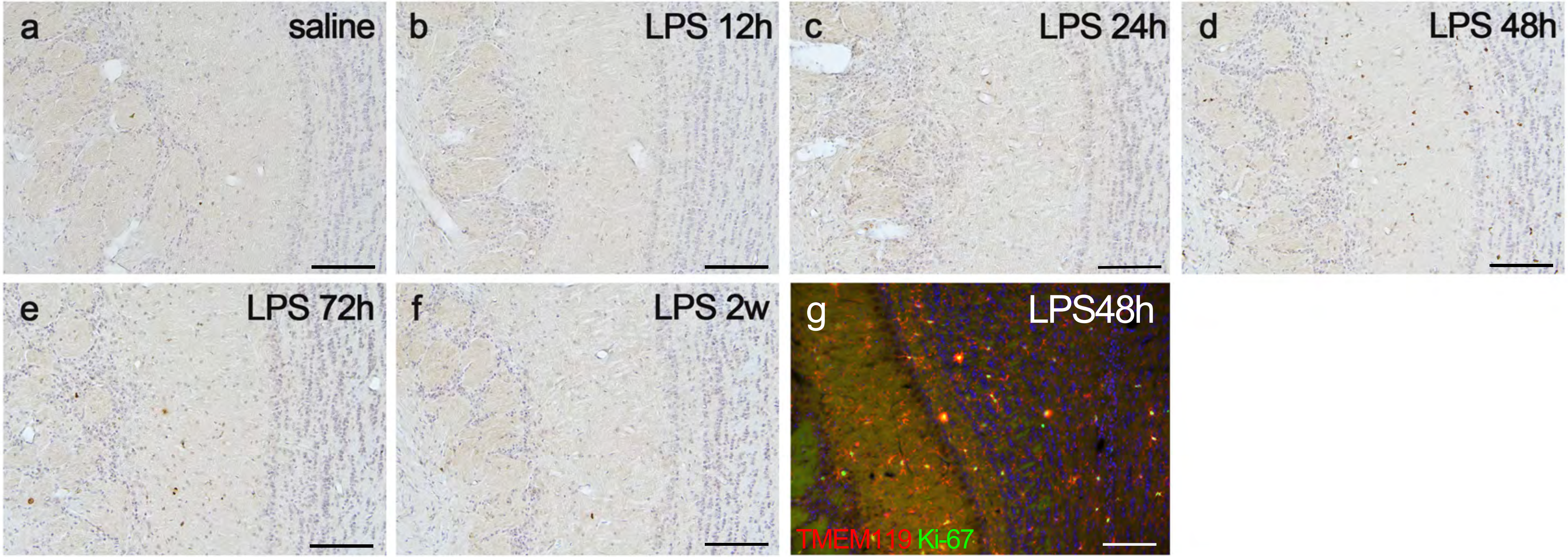
a
saline
b
LPS 12h
c
LPS 24h
d
LPS 48h
e
LPS 72h
f
LPS 2w
g
LPS48h
TMEM119 Ki-67
